# Integrating Maternal and Children's Oral Health Promotion into Nursing and Midwifery Practice- A Systematic Review

**DOI:** 10.1371/journal.pone.0166760

**Published:** 2016-11-23

**Authors:** Reham Abou El Fadl, Mitch Blair, Sondus Hassounah

**Affiliations:** 1 MPH, School of Public Health, Imperial College of London. Pediatric Dentistry and Dental Public Health Department, Faculty of Dentistry, Ain Shams University, Cairo, Egypt; 2 Department of Paediatrics, River Island Academic Centre for Paediatrics and Child Health, Imperial College London, London, United Kingdom; 3 WHO Collaborating Centre for Public Health Education and Training, Department of Primary Care and Public Health, Imperial College London, London, United Kingdom; TNO, NETHERLANDS

## Abstract

**Background:**

Globally, oral diseases contribute to major disease problems and oral health disparities persistently exist amongst vulnerable population groups. Two contributory factors to these challenges are the shortage of dental practitioners and the characteristic separation between the medical and dental professions. Nurses and midwives, in particular, are in a potentially excellent position to assist in basic oral health services such as dental health education and intraoral screening. We aimed to assess the effectiveness of integrating promotion of oral health of young children and their mothers into nursing and midwifery practice.

**Methods and Findings:**

Seven electronic databases including CENTRAL, EMBASE, MEDLINE, GLOBAL HEALTH, CINHAL, Scopus, and Web of Science were systematically searched whereas conference proceedings and theses were retrieved via PROQUEST. Only randomized, non-randomized trials and observational studies on preventive oral health programs delivered by nurses or midwives in healthcare settings or through home visits were included. Two investigators reviewed full-text articles independently to decide on eligibility for inclusion. Quality assessment was done using Cochrane tool for risk of bias for randomized trials and Downs and Black assessment tool for all other studies. Out of 3162 retrieved records, twenty one trials on oral health interventions incorporated into standard nursing practice were reviewed. Eighteen programs reported significant positive outcomes including reduction in caries experience, better oral hygiene and dietary habits and increased rates of dental visits amongst young children as reported by their caregivers.

**Conclusions:**

Incorporating oral health promotion into nursing practice is a promising initiative for reducing oral health disparities by contributing to a downward trend in caries experience and increased access to dental care especially amongst the poor disadvantaged communities.

## Introduction

Oral health is considered an integral part of general health. The World Health Organization (WHO) defines oral health as “a state when an individual is free of chronic oro-facial pain, oral sores or cancer, craniofacial defects such as oral clefts, gum diseases, dental decay, tooth loss or any other disorders affecting oro-dental tissues”. [1] The effects of oral diseases go far beyond the oral cavity and are strongly linked to major chronic disease problems such as obesity, cardiac diseases, diabetes, and respiratory infections. [2, 3]

There is mounting evidence that maternal oral health status and oral hygiene practices have a significant influence on both children’s general and oral health. [4] Pregnant women are more liable to experience oral problems due to various hormonal changes and fluctuations in intraoral flora; nearly 40% of pregnant women suffer from some form of periodontitis and up to 10% may develop pregnancy oral tumors especially amongst the under-privileged. [5] Despite that, it is not uncommon that pregnant women would not seek care even when having oral problems. [6]

An infant’s risk to experience dental decay due to early acquisition of transmitted microorganisms is strongly associated with mother’s high titres of cariogenic bacteria. [5, 7] Unfortunately, oral health is usually a much-neglected aspect of perinatal care and there is a lack of appreciation amongst caregivers that primary teeth do really matter. Regular dental visits for receiving oral care are not the norm in childhood and adolescence. [8] This is mainly attributed to inadequate financial resources, shortage or mal-distribution of dental personnel, in addition to the absence of integration with other non-dental healthcare professionals (HCP). [9] Compounding the situation is the diminished oral health literacy due to lack of public awareness of the impacts of oral health on the well-being throughout the course of life. [10] Moreover, societal and cultural norms and the erroneous beliefs that oral health is isolated from general health altogether contribute to underutilization of the dental services. [11]

In 2009 the WHO 7^th^ global conference has advocated the integration of dental care into primary healthcare services and reliance on the collaborative work of a diverse array of HCP. This integrative strategy rests on the premise that a cluster of modifiable risk factors such as diet and smoking contribute to oral and non-communicable diseases (NCD) together. [11]

Incorporating an oral health component into prenatal services necessitates coordination between dental providers and “front-line” HCP such as pediatricians, family physicians, midwives, and nurse practitioners.[12] Nurses and midwives are, in particular, ideally positioned to positively contribute to promoting oral health status of children and mothers and expand their access to preventive dental care especially in deprived populations. Maternity and pediatric nurses can routinely provide expectant mothers with oral health counseling before and after childbirth. They can also play a pivotal role in identifying at-risk mothers or children by performing oral screening and risk assessment to inform subsequent referral to dentists for dental treatment. [13, 14]

Accordingly, we conducted this review to generate evidence from randomized, non- randomized clinical trials and observational studies on the effectiveness of integrating oral health promotion into basic services delivered by nurses and midwives to childbearing women and very young children.

## Methods

### Search Strategy

In our systematic review, we followed the Preferred Reporting Items for Systematic Reviews and Meta-Analyses (PRISMA) guidelines. **(S1 File)** [15] A rigorous search of seven electronic databases including EMBASE, MEDLINE and GLOBAL HEALTH via OVID, CINAHL via EBSCO, Scopus, Web of Science and CENTRAL was performed from inception to 1st of July, 2015. In order to capture grey literature a supplementary search for conference proceedings and theses was undertaken via ProQuest. **(S2 File)** Reference lists of all recovered studies were also explored for potentially relevant literature. We expanded our search terms to ensure that any relevant publications were not overlooked. The search was not time bound or restricted by language and included the following combination of MeSH terms (Medical Subject Headings) and keywords: [‘oral health’ OR ‘dental health’ OR ‘dental care’ OR ‘oral hygiene’ OR ‘oral care’ OR ‘dental caries’ OR ‘gingival disease’] AND [‘health promotion’, OR ‘dental health education’, OR ‘oral health education’ OR ‘preventive dentistry’] AND [‘nurses’, OR ‘midwives’ OR ‘midwifery’ OR ‘nursing ‘ OR ‘health visitor’ OR ‘home visitor’].

### Study Selection

**Fig 1:**
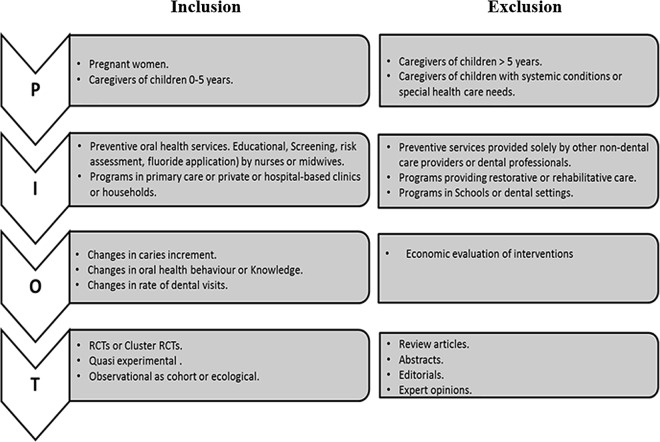
Inclusion and Exclusion criteria

Randomized controlled trials (RCTs), cluster RCTs, ‘quasi-experimental’ and observational studies were included. Only studies assessing the effectiveness of primary preventive oral health programs for very young children (0–5 years old) or childbearing women where nurses or midwives were amongst the delivery team were reviewed. A range of outcomes were set for this research whereby for a study to be included in the review it should assess changes in one or more of the following: i) parental oral health knowledge; ii) adherence to oral hygiene measures; iii) caries experience, iv) rates of dental attendance. Studies were excluded if they were designed to target caregivers of children in other age groups or those with special health care needs or any systemic problems. Programs incorporating restorative or rehabilitative dental care and those that were solely provided by lay health workers or by other non-dental healthcare professionals (HCP) such as pediatricians, obstetricians, family physicians, or by any active members of dental care team such as dentists or dental nurses or therapists were not included either (**Fig 1).**

Initially, all retrieved citations were checked for any duplicates which were then discarded. The remaining studies were then screened for relevance based on titles and abstracts. For studies which could not be adequately excluded based on the title or abstracts, full text was obtained and reviewed to assess for eligibility. All articles were independently screened by two authors (RA and SH) and whenever necessary any disagreements on inclusion or exclusion of studies were resolved through discussion until consensus was reached.

### Data extraction and quality assessment

In our review, data was abstracted and recorded in a data extraction form adopted from other Cochrane systematic reviews [16]. General information was extracted on study characteristics (year of publication, study design, setting, duration, and study location), population characteristics (age, gender, number of participants at enrollment and follow up). Furthermore, detailed description of the interventions, any theoretical frameworks adopted in their design, delivery personnel, the main outcome measures and findings were all recorded.

We opted to use two different critical appraisal tools based on study design; RCTs were assessed using Cochrane Collaboration’s tool for assessing risk of bias [17] whereas non-randomized trials were assessed using Downs and Black assessment tool. [18] For each RCT, seven domains (random sequence generation, allocation concealment, blinding of participants and personnel, blinding of outcome assessors, incomplete outcome data, selective outcome reporting and other bias) were addressed. A judgment of ‘Low risk’, ‘High risk’, or ‘Unclear risk’ of bias was assigned for each item in the tool alongside with descriptive information justifying this judgment

On the other hand for each non-randomized trial a total numeric score was given for 27 items categorized into 4 sections: reporting (10 items), external validity (3 items), internal validity (bias: 7 items, confounding 6 items) and power of study (1 item). For each question answers scored 1 for “yes” responses or 0 for “no” or “unable to determine” responses. Only for one item in the reporting section: *“Are the distributions of principal confounders in each group of subjects to be compared clearly described*?*”* scoring ranged from 0 for “no”, 1 for “partially” to 2 for “yes” responses. [18] Two modifications were done such that one item on reporting any adverse events was removed since all reviewed interventions were non-invasive preventive. In addition, concerning the power of calculation for sample size, studies were scored 0 or 1 based on whether or not the calculation was performed. [19] The total Down and Black score (D&B) ranged from 0 to 27 and the following cut points have been used to categorize studies by quality: excellent (26–27), good (20–25), fair (15–19) and poor (≤14)

## Results

**Fig 2:**
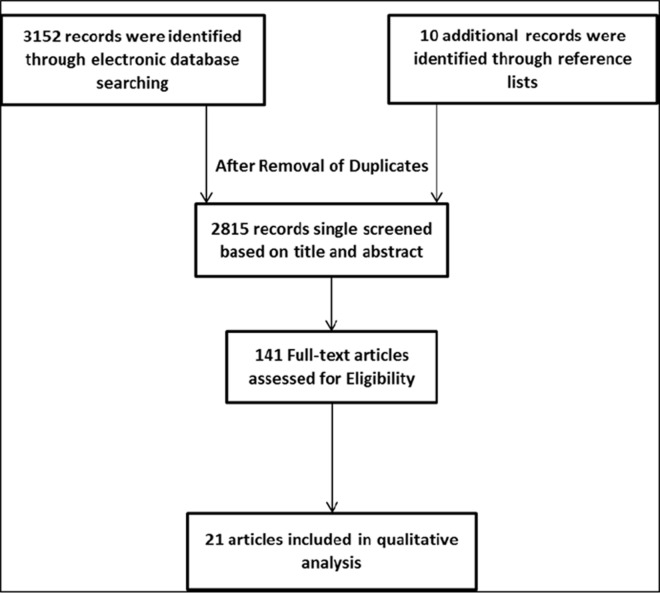
PRISMA flow Chart

Of the 3162 records initially retrieved, 141 were eligible for full text screening after removal of duplicates and screening titles and abstracts. Only twenty one studies [20–40] met the inclusion criteria; those were published between 1993 and 2015 **(Fig 2).** The main reasons for exclusion were that studies did not fit the set inclusion criteria concerning one of the following: i) the study design, ii) the delivery personnel of the interventions iii) the outcomes used to measure their effectiveness.

The included studies were conducted across 7 countries such that eight programs were implemented in the US, five in the UK, two in Brazil, one in Iran, Belgium, Australia, and India. On reviewing the studies, we found that two retrieved RCTs [26, 27] in Brazil and another two in Iran [32, 33] addressed the same intervention but with different outcomes or over different follow-up duration. **Table 1**

**Table 1:** Characteristics of the included studies

**Author**	**Year**	**Country**	**Target population**	**Study setting**	**Study design**	**Risk of bias /D&B scores**
Bentley etal.	1993	UK	0–2 years(n = 3165)	households	quasi-experimental pre-test/post-test design	D&B score = 10
Biordi et al.	2015	USA	2 years(n = 4360)	households	quasi-experimental pre-test/post-test design	D&B score = 11
Braden et al.	2013	Belgium	Newborn(0–10 months)(n = 2137)	well-baby clinics & households	quasi-experimental with equivalent comparison gps.	D&B score = 20
Brickhouseet al.	2013	USA	6–36 months(n = 432)	households,rural settings	historically-controlled non-randomized study	D&B score = 20
Chaffee et al.	2013	Brazil	New-born (n = 715)	public primary health care centers	Cluster RCT	low risk
Cibulka et al.	2011	USA	Pregnant women 18–39 yrs.(n = 170)	hospital-based clinic, urban	RCT	high risk
Feldens et al.	2007&2010	Brazil	New-born (n = 500)	households,urban	RCT	high risk
Hallas et al.	2015	USA	New born (n = 94)	hospital- based clinic, urban	RCT	high risk
Kressin et al.	2009	USA	Children 6months to<5yrs(n = 1087)	outpatient clinic, academic medical center	quasi-experimental with equivalent comparison gps.	D&B score = 20
Achembong et al.	2014	USA	Children 0–3.5 years(n = /920, 505)	primary care medical offices	Ecologic observational	D&B score = 16
Mattheus DJ	2014	USA	Children 6–9 month(n = 100)	Pediatric private clinic	RCT	high risk
Mohebbi et al.	2009& 2012	Iran	Children 12–15 month(n = 242)	Public health centers	RCT	low risk
Nair et al.	2009	India	Children <6 yrs.(n = 2708)	Anganawadi rural	quasi-experimental pre-test/post-test design	D&B score = 13
Neumann et al.	2011	Australia	Children 12–24 month(n = 1085)	local community centers, rural	RCT	high risk
Whittle et al.	2008	UK	Children 8 months (n = 501)	households	RCT	high risk
**Wilson et al.**	2013	USA	Children 6–36 months (n = 104)	households,rural	quasi-experimental pre-test/post-test design	D&B score = 20
**Yuan et al.**	2007	UK	Children 0-2and 3–5 yrs.	households, rural &urban	quasi-experimental with non-equivalent comparison gp.	D&B score = 13
**Kowash et al.**	2000	UK	Children 8 months(n = 228)	households	RCT	unclear risk
**Davies et al.**	2005	UK	Children 8 months(n = 1545)	health districts (primary care groups)	RCT	high risk

Of the 21 studies twelve were either RCTs or cluster randomized trials. [24–28, 32, 33, 35, 36, 39, 40] Four trials, however, were “quasi-experimental” with a pre-test/post-test design [20, 21, 34, and 37], two had equivalent [22, 29] and one had non-equivalent comparison group^.^ [38] One included study was historically controlled non-randomized [23] and another was ecologic. [30] **Table 1**

Some diversity in studies’ settings was recognized such that five programs were based in public health or municipal or medical academic or women and infant care centers [21, 24, 32, 33, and 40], two studies were conducted in hospital based clinics, one in a private clinic [31] and one in an “Anganwadi” [34] a care setting typical to India. Seven home visiting programs [20, 23, 26&^27^, ^36^, ^37^, ^38^, ^39^] were reviewed as well whereas one intervention included both home visits and consultations at well-baby clinic. [22] Two studies [21, 38] reported including both urban and rural settings in their recruitment, five [23, 30, 34, 35, 37] were restricted to rural sites and three [25, 26, 28] to urbanized or metropolitan areas. **Table 1**

### Participants

The size of the trials varied between 94 to 4360 participants and only seven trials reported conducting the power calculations to decide on their sample sizes.[24,25,26&^27^,^28^,^32^&^33^,^36^,^40^] Only one program [25] targeted pregnant women while all the other interventions focused on children’s oral health from birth till the age of five. In total 4846 caregivers participated in ten RCTs, 2569 of which were in the intervention arm. In the “Into the Mouths of Babies Program” (IMBP) in North Carolina, panel data from oral health surveillance system was used for 946, 911 kindergarten children. [30] In all included trials, the age range of the enrolled children ranged from zero to five years; only five programs [20, 22, 24, 26&^27^, ^28^] started immediately after childbirth whereas one study [38] divided the participants into two different age groups(0–2 and 3–5 years). **Table 1**In some of the included studies [21, 23, 30, 31, and 37] the recruited children were already enrolled in existing programs delivering early childhood healthcare services such as Medicaid, Early Head start, or Women Infant Care Programs. As revealed from extracted data, the majority of the reviewed interventions targeted vulnerable underserved low-income populations.[20,21,23,25,26&^27^,^29^,^32^&^33^,^35^,^37^,^40^]

### Interventions

Eight trials synchronized children’s oral health promotion services with “well-child” or vaccination visits. [21, 22, 29–33, 40] In all the reviewed interventions, oral health education was a basic component provided by adopting various approaches. Some of the participants viewed didactic videos [25, 28, and 37]; Brickhouse et al. [23] used oversized models to demonstrate teeth cleansing techniques, other interventions disseminated information via leaflets, pamphlets, or booklets and Kressin et al. [29] used role-play exercises to deliver the message. In ten trials oral health kits including toothbrush, paste and feeding cups were given to enrollees in the intervention groups. [28, 35, 36, 38] Fluoride varnish application by nursing staff was incorporated in four programs. [21, 23, 30, and 31] For enhancing dental attendance, participants were either scheduled for dental check-ups or received dental registration vouchers and or dentists’ contacts. **Table 2**

**Table 2:** Intervention description and summary of outcomes

Study author	Description of intervention	Delivery Team	Measured Outcomes		
			Caries experience	Oral health knowledge & practice	Utilization of dental care
Bentley et al.	Health education in single home visit + dental referral	Health Visitors	-	-	0
Biordi et al.	Oral screening, fluoride application & counseling in 3 home visits over 14 months	Dietitian & pediatric Nurses	+	-	+
Braden et al.	Oral health counseling in 11sessions and 3 home visits	Nurses & physicians	0	0	0
Brickhouse et al.	Fluoride application and counseling / one time intervention	Community health nurses & pediatric Nurses	-	-	+
Chaffee et al.	Nutritional counseling	Nurses & Physicians	0	-	-
Cibulka et al.	Educational video and referral for checkup/ one time intervention	Advanced practice nurses	-	+	+
Feldens et al.	Breast feeding & nutritional counseling at home visits on monthly basis for 6 months & at 8,10,12 months	Health visitors	+	+	-
Hallas et al.	Educational video & oral health kit/one-time intervention	Pediatric nurse practitioner students	0	0	-
Kressin et al.	Role play exercises & educational brochure in one time brief intervention	Pediatricians and clinic nurses	+	-	-
Achembong et al.	Dental screening, fluoride varnish & counseling over 6 medical visits (IMBP)	Physicians, registered nurses & nurse practitioners	+	-	+
Mattheus DJ		Pediatric nurse practitioners	-	+	-
Mohebbi et al.	One counseling session, educational brochure &\ phone calls reminder twice a month for 6 months	Vaccination staff	+	+	-
Nair et al.	Oral health counseling in one session	Junior public health nurses & anganwadi workers	-	+	-
Neumann et al.	Counseling session & oral health kit/one-time intervention	Maternal child health nurses	+	-	-
Whittle et al.	Educational leaflet, oral health kit & diet record sheet in 2 home visits over 12 months	Health visitors	0	-	-
Wilson et al.	Didactic or family centered video sessions & a video copy in home visits over 8–10 weeks	Head Start home visitors	-	+	-
Yuan et al.	Counseling, oral health kit, dental registration voucher & lists of dentists in home visits at 7 weeks, 8 months and 18 months	Community- based nurses	-	-	+
Kowash et al.	Oral health advice in home visits over 3 years	Outreach pediatric nursing sisters	+	+	+
Davies et al.	Counseling, oral health gifts & written pictorial instructions over 2 years/follow up at 8 12–15,18, 26 & 32 months	Health visitors or practice nurses	+	-	-

(+): Significant improvement in the measured outcomes in the intervention group compared to control group

(0): Insignificant difference in the measured outcomes between the intervention and control group

(-): no measurement reported

Nurses played a pivotal role in all the programs yet the structure of the delivery team varied. Interventions were implemented either entirely by nursing staff [23, 25, 28, 31, 35, and 38] or in partnership with other non-dental personnel such as pediatricians, nutritionists or vaccination staff. [22, 24, 29, 30, 32&^33^] No studies integrating oral health promotion into midwifery practice were identified. **Table 2**

### Outcomes

Thirteen reviewed studies considered caries increment as an outcome measure, three of which revealed insignificant effects on caries prevalence among children. [22, 24, 36] In all other trials [21, 26&^27^, ^29^, ^30^, ^32^&^33^, ^35^, ^39^, ^40^] the tested interventions yielded significant reduction in caries experience among the participants. According to Hallas et al. [28], however, they failed to assess the effectiveness of the delivered program as planned due to the high attrition rate in the study. **Table 2**Regarding the impact on oral health knowledge and practices, as revealed in six reviewed programs [21, 25, 26&^27^, ^31^, ^37^, ^39^] the frequency of oral hygiene measures and dietary habits significantly improved amongst participants post-intervention. Eight trials considered rates of dental attendance as a measure of performance. In three clinic-based studies [21, 25, 30] the authors reported that delivering the interventions has contributed to increased likelihood of subsequent dental visits amongst the participants whereas in one program in Belgium higher rates of dental attendance were observed among controls. [22] Moreover, the rates of dental registration amongst children were significantly higher five month following two home visiting campaigns. [38, 39] **Table 2**

### Quality of studies

**Fig 3:**
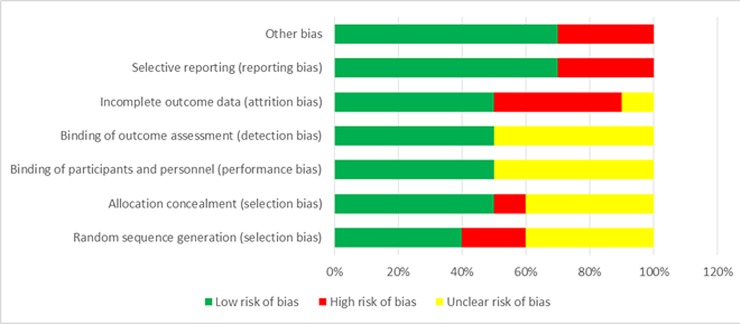
Risk of bias graph for RCTs

Our assessment for the quality of the RCTs revealed that only two of them [24, 32&^33^)] were judged to be at low risk of bias for all domains. Across the domains relating to performance and detection bias, all of the trials scored low or unclear, whereas in terms of selection bias one trial [25]scored high in both sequence generation and concealment and another [26&^27^] scored high only for sequence generation. Attrition bias was the highest source of bias, being scored as high for four trials (**Fig 3).** In the study in Australia, [35] authors reported a potential towards response bias as participating families tend to care generally about health and attend maternal and child care centers frequently. In another program [36] failure to restrict information to controls might have contributed to cross contamination.

On the other hand, according to Downs and Black checklist, of the non-randomized studies, we rated four [22, 23, 29, and 37] as of good quality; four were poor quality and one study scored as fair. [30] The minimum score, 10, was assigned to Bentley et al. [20] and the maximum, 20, to the studies of Branden.et al, [22] Brickhouse. et al. [23], Kressin et al. [29] and Wilson et al. [37] **Table 1**

## Discussion

Despite strategies undertaken to ameliorate the social disparities in oral health worldwide, oro-dental problems particularly amongst young children with either social or medical vulnerabilities continue to be a major issue. [41]

To the best of our knowledge, this is the first attempt to systematically review the effectiveness of interventions delivered by non-dental nursing personnel to promote oral health of women of childbearing age or very young children. There is moderate evidence that such inter-professional collaboration and focus, especially when embedded into an existing childcare system, results in improved child and to a lesser extent maternal oral health status and reduces oral health disparities within disadvantaged populations. Integrating oral care interventions into nursing practice can be feasibly implemented to serve different community groups across a multitude of locations ranging from primary care and maternal child health centers, hospital-based clinics to home settings.

Although the significance of maternal oral health has been reiterated in many practice guidelines, [42] all the included trials, except for one program [25] aimed to improve the oral health of only new-born and preschoolers indicating some bias towards children. The intimate association that exists between maternal and child oral health would suggest a greater degree of effort is needed to create and evaluate community-based programs for adapting the prenatal care model to include dental care as a routine service for pregnant women.

Our review also showed that it is possible to employ staff from a wide range of specialties and levels of education to deliver interventions targeting oral health promotion successfully. According to Kowash et al. [39] dental health services delivered by a dental therapist and a pediatric nurse were quite comparable and in another program [21] a pediatric nurse practitioner was qualified enough to train the delivery team on dental screening and fluoride application. Hence, it is highly advocated to incorporate basic knowledge and guidelines on oral disease prevention into the existing undergraduate and postgraduate curricula of non-dental HCP, familiarize and appropriately train staff about clinical and referral pathways. This would help in capitalizing their commitment to promoting maternal and children oral health and permit the transfer of some tasks such as oral risk assessment and counseling from dental health professionals, in turn allowing them to focus on more specialized tasks.

Nurses in particular are capable of early provision of key primary preventive oral services as well as keeping the momentum going for maintaining their delivery. This can be optimally done through integrating dental care into well-child primary care sites as stated by Biordi et al. [21] to take advantage of the regular appointments routinely scheduled for children’s general health such as for vaccination. A pool of children and their caregivers from all social backgrounds could be then captured and a dental home could be initiated for all children as early as one year of age when dental visits are rare. Thereafter, participants receiving preventive services could be entered into a tracking program for assessing the effects of the interventions and facilitating dental referrals of at-risk patients. This was revealed, in the IMBP [30] where caries experience of enrollees and numbers of their dental visits were estimated annually over nine years.

With regards to minority, low-income and marginalized groups per se; it has been well-known that financial barriers, the unavailability of dental care in certain areas, lack of transportation and decline in dental workforce are the main reasons for their limited access to oral health services. [9] Adopting home visiting models could pave the way for community nurses [43] to outreach these populations, in particular, in order to close the gaps in dental care by improving their oral health literacy and referring them to appropriate treatment services. This has been verified in three reviewed programs [37, 39, and 40] where health visiting brought about significant improvement in the set health-related outcomes.

Based on a series of theories, socio-cultural factors are known to have an impact on individuals’ perceptions of their health status, their lifestyles and health-related practices as well as their care-seeking behaviors. Amongst the social determinants of health, socioeconomic position and social support are the most influential. [44] In Belgium, though “Smile for Life” was a multi-component, theory-based program [22] where most of the fundamentals for a health promotion intervention were considered yet the encountered impact was quite limited. As elucidated by the authors, half of the enrolled mothers were highly educated hence the program’s goals might have been met primarily amongst the socially and educationally disadvantaged groups with highest unmet oral health needs. In support of this, ten of the reviewed programs [21, 23, and 25, 28–30, 37–40] targeted underserved low-income populations and all proved highly effective.

In acknowledgment of the pivotal role of social support, in one home visitation program [37] the researchers successfully employed what they called”Pass it on” strategy to benefit from mobilizing social cohesion within one community for rapid reinforcement of positive behaviors. This was facilitated by the common norms, mutual trust and daily close interactions especially amongst the poor. [45]

As regards to the cultural context, knowing that beliefs and values are quite diverse among different population groups even, within the same country, it is quite crucial to incorporate such beliefs and values in health-related interventions. [46] Nurses are ideally positioned to develop trusting relationships with mothers especially in rural communities and among the underprivileged. As a result, they tend to be highly knowledgeable about their people’s views [47] and thus can effectively help in designing culturally acceptable and appropriate interventions which can eventually improve maternal and children’s oral health status.

Since the responsiveness of any targeted population to a health intervention is of paramount importance for ensuring the fidelity of its implementation, much emphasis need to be put on its content, frequency and duration of delivery. [48] Generally, the delivery strategy for health counseling is a key consideration. Our findings suggest that using multi-faceted approaches could have significant positive impact on caregivers’ oral health behaviors. Combining different pedagogical tools such as video presentations [24, 37] and take-home leaflets printed in the language of target population with dental kits of toothpaste and brushes [32, 33, and 40] would trigger an immediate translation of the gained knowledge into improved oral health practices.

As revealed, in our review the longest duration for an intervention was three years in only two studies. [22, 39] Typically, community-based programs need a minimum of five years to detect any impacts as individuals rarely modify lifetime habits over a short period of time. [49] Regarding the frequency, in three trials [37, 38, and 39] it was asserted that repeating oral health counseling created a platform for advancing caregivers’ oral health literacy and empowered them to engage in positive behaviors for themselves and their children as well. In one reviewed study, however, according to the authors, the lack of intensity of their one-time program was a limitation, which yielded modest insignificant effects post-intervention. [35] Consequently, it could be deduced that to attain the goals of any oral health programs an adequately frequent contact with the target groups need to be maintained over a long period of time.

Although a full economic evaluation is outside the scope of our review, many authors described their interventions as of low cost especially when incorporated into existing routine childhood health services. Drawing on this finding, such programs are potentially replicable in high, middle and low income countries, where resource allocation varies considerably. [50]

### Limitations and implications for future research

Within the included studies, one drawback which didn’t escape our notice was the lack of any theoretical basis in their design. In our review only three trials [22, 32&^33^, ^35^] referred to using a theory to inform their interventions and none of them was explicit as to how this was performed. Thus, undoubtedly, any future programs for reinforcing positive health-related behaviors need to carefully apply a theoretical framework that could address both internal and external factors that might influence the individuals’ attitudes and practices. Moreover, pilot work was not conducted to inform the design of the programs in any of the included studies prior to intervening. However, according to the Medical Research council guidelines, piloting any complex interventions is quite crucial for assessing their acceptability, identifying the barriers and facilitators for implementation and evaluating the feasibility of embarking on a large scale within a defined context especially for newly “emerging” interventions. [51]

Though RCTs are the gold standard for public health interventions [52] yet in community based settings it might be infeasible and impractical to implement them. [53] Subsequently, we did not limit the included studies to RCTs since considering other sources of evidence as non-randomised trials or observational studies could provide initial insights on feasibility of an intervention especially when there is consistency in findings across programs by different investigators in different settings. [54] While this research succeeded to identify twenty one studies with potentials for improving children’s oral health through partnership with nursing personnel, yet there is still scarcity in evidence from high quality low bias RCTs. Thus, in future studies, researchers need to describe in details the methods of randomization and allocation concealment employed and clearly address blinding at different levels to minimize the risk of bias and ensure that high quality evidence is generated.

One limitation in our systematic review was that narrative synthesis was undertaken rather than a meta-analysis. However, this was done due to heterogeneity across studies arising from differences in sample characteristics (number of participants and gender and age distributions), length of follow-up and study settings as well as the lack of uniformity in the design of the included studies, the description of the interventions, their delivery team and measurement of their outcomes.

Though the search process was quite robust as it included seven electronic databases and grey literature and captured studies from countries all over the world targeting different population groups yet qualitative research was not part of the review such that studies assessing both nurses’ and midwives’ perceptions and views on their participation in improving oral health and caregivers’ experiences with this type of interventions were excluded. We, thus, recommend reviewing relevant qualitative research to gain more insight into the views of providers regarding their self-efficacy and willingness to engage in such activities as well as the level of acceptability of these interventions amongst target populations and their readiness to comply.

## Supporting Information

S1 File PRISMA checklist. (DOC)

S2 File Search Strategy. (DOCX)
