# Supplementary material for: Integrating Maternal and Children's Oral Health Promotion into Nursing and Midwifery Practice- A Systematic Review
Source: PLoS One. 2016 Nov 23;11(11):e0166760. doi: 10.1371/journal.pone.0166760 (PMC5120808; doi:10.1371/journal.pone.0166760)
Supplement: S2 File — (DOCX) [file pone.0166760.s002.docx]

**S1 File Supporting Information Search strategies for literature review**

**Database: EMBASE via OVID**

**Search strategy:**

1. dental health/

2. mouth hygiene/

3. dental caries/

4. periodontitis/ or dental care/ or periodontal disease/

5. gingiva disease/

6. ((oral adj2 care) or (mouth adj2 care) or (dental adj2 care) or (teeth adj2 care)).mp.

7. ((oral adj2 hygiene) or (mouth adj2 hygiene) or (dental adj2 hygiene) or (teeth adj2 hygiene)).mp.

8. ((oral adj2 health) or (dental adj2 health) or (teeth adj2 health) or (mouth adj2 health)).mp.

9. (gingival diseas* or periodont*).mp.

10. (dental caries or dental decay).mp.

11. or/1-10

12. preventive dentistry/

13. health promotion/

14. dental health education/

15. (health adj2 (promot* or advi?e or educat* or practic* or improv*)).mp.

16. (prevent* adj2 oral).mp.

17. (prevent adj2 dent*).mp.

18. Or/12-17

19. midwife/

20. nurse midwifery/ or nurse midwifery education/ or neonatal nurse practitioner/ or nurse midwife/ or pediatric nurse practitioner/ or nurse practitioner/ or pediatric nurse/ or nurse/

21. health visitor/

22. health care personnel/

23. ((nurs* adj2 educat*) or (midwi* adj2 educat*) or nurs*adj2 train or (midwi* adj2 train*) or (nurs* adj2 practic*) or (midwi* adj2 practic*)).mp.

24. nursing staff/ or nursing/ or community health nursing/ or nursing education/ or perinatal nursing/ or newborn nursing/ or nursing practice/ or obstetrical nursing/ or pediatric nursing/

25. (health visit* or home visit*).mp.

26. or/19-25

27. 11 and 18 and 26

**Database: MEDLINE via OVID**

**Search** **strategy:**

1. Oral Health/

2. Dental Caries/

3. Dental Care/

4. Dental Care for Children/

5. Periodontal Diseases/

6. Gingival Diseases/

7. Oral Hygiene/

8. Periodontitis/

9. ((oral adj2 care) or (mouth adj2 care) or (dental adj2 care) or (teeth adj2 care)).mp.

10. ((oral adj2 health) or (dental adj2 health) or (teeth adj2 health) or (mouth adj2 health)).mp.

11. ((oral adj2 hygiene) or (mouth adj2 hygiene) or (dental adj2 hygiene) or (teeth adj2 hygiene)).mp.

12. (dental caries or dental decay).mp.

13. (gingival diseas* or periodont*).mp.

14. or/1-13

15. Preventive Dentistry/

16. Health Promotion/

17. Health Education, Dental/

18. (health adj2 (promot* or advi?e or educat* or practic* or improv*)).mp.

19. (prevent adj2 oral).mp.

20. (prevent adj2 dent*).mp.

21. 0r/15-20

22. Midwifery/

23. Nurses/

24. Education, Nursing/ or Nursing/ or Public Health Nursing/ or Neonatal Nursing/ or Pediatric Nursing/ or Maternal-Child Nursing/ or Community Health Nursing/ or Primary Care Nursing/ or Obstetric Nursing/ or Nursing Staff/

25. Health Personnel/

26. ((nurs* adj2 educat*) or (midwi* adj2 educat*) or nurs*adj2 train or (midwi* adj2 train*) or (nurs* adj2 practic*) or (midwi* adj2 practic*)).mp.

27. (health visit* or home visit*).mp.

28. Or/22-27

29. 14 and 21 and 28

**Database: GLOBAL HEALTH via OVID**

**Search strategy:**

1. dental health/

2. oral health/

3. dental caries/

4. tooth diseases.sh.

5. (periodontal diseases or gingivitis).sh.

6. mouth diseases.sh.

7. periodontitis/

8. ((oral adj2 care) or (mouth adj2 care) or (dental adj2 care) or (teeth adj2 care)).mp.

9. ((oral adj2 health) or (dental adj2 health) or (teeth adj2 health) or (mouth adj2 health)).mp.

10. ((oral adj2 hygiene) or (mouth adj2 hygiene) or (dental adj2 hygiene) or (teeth adj2 hygiene)).mp.

11. (gingival diseas* or periodont*).mp.

12. (dental caries or dental decay).mp.

13. Or/1-12

14. health promotion/

15. preventive dentistry/

16. (health adj2 (promot* or advi?e or educat* or practic* or improv*)).mp.

17. (prevent* adj2 oral).mp.

18. (prevent* adj2 dent*).mp.

19. Or/14-18

20. (midwives or health care workers).sh.

21. nurses/

22. nursing/

23. ((nurs* adj2 educat*) or (midwi* adj2 educat*) or nurs*adj2 train or (midwi* adj2 train*) or (nurs* adj2 practic*) or (midwi* adj2 practic*)).mp.

24. (health visit* or home visit*).mp.

25. healthcare work*.mp.

26. OR/20-25

27. 13 and 19 and 26

**Database: SCOPUS**

**Search strategy:**

("oral health" OR "dental health" OR "oral care" OR "dental care" OR " oral hygiene" OR "dental caries" OR "gingival disease" )

AND (health promotion OR health education OR prevention)

AND ( nurses OR midwives OR midwifery OR nursing OR health visitors OR home visitors ) )

**Database: WEB of SCIENCE**

**Search strategy:**

(Search Refined by: Research areas: (Health care sciences services OR Nursing OR Pediatrics OR Dentistry Oral surgery medicine OR Behavioural Sciences) AND Databases: (Web of Science Core Collection)

#1 ((oral or mouth or dental or teeth) AND (hygiene or health or care))

#2 (("dental caries" or” dental decay “or” gingival diseas*" or” periodont*"))

#3 #1 or #2

#4 ((teeth or dental or mouth or oral or health) AND (advis* or promot* or educat* or improv* or instruct*))

#5 ((prevent*) AND (dental caries or dental decay))

#6 ((prevent*) AND (gingival diseas* or periodont*))

#7 ((prevent*) AND (oral diseas* or dental diseas*))

#8 #4 or #5 or #6 or # 7

#9 ((nurs*) AND (practic* or educat* or train*))

# 10 ((midwi*) AND (practic* or educat* or train*))

# 11 ((health visit* or home visit*))

# 12 ((healthcare) AND (workers or personnel))

# 13 # 9 or # 10 or # 11 or # 12

#14 ((child* or infan*or preschool or newborn*))

# 15 ((pregnan* or prenatal*or antenatal or perinatal*))

# 16 # 14 OR # 15

#17 # 3 AND #8 AND # #13 AND # 16

**Database: The Cochrane Central Register of Controlled Trials (CENTRAL)**

**Search strategy:**

#1 MeSH descriptor: [Oral Health] explode all trees

#2 MeSH descriptor: [Oral Hygiene] explode all trees

#3 MeSH descriptor: [Dental Care] explode all trees

#4 MeSH descriptor: dental care for children this term only

#5 MeSH descriptor: dental caries this term only

#6 MeSH descriptor: gingival disease this term only

#7 MeSH descriptor: periodontal disease this term only

#8 (dental near/2 care) or (mouth near/2 care) or (oral near/2 care) or (teeth near/2 care)

#9 (mouth near/2 health) or (oral near/2 health) or (dental near/2 health) or (teeth near/2 health)

#10 (mouth near/2 hygiene) or (oral near/2 hygiene) or (teeth near/2 hygiene) or (dental near/2 hygiene)

#11 (dental caries or dental decay)

#12 (gingival diseas*) or (periodont*)

#13 ( #1 or #2 or #3 or #4 or #5 or #6 or #7 or #8 or #9 or #10 or #11 or #12)

#14 MeSH descriptor: [Health Promotion] explode all trees

#15 MeSH descriptor: [Health Education, Dental] explode all trees

#16 MeSH descriptor: [Preventive Dentistry] explode all trees

# 17 (prevent* near/2 oral) or (prevent* near/2 dent*)

# 18 (health near/2 promot*) or (health near/2 advis*) or (health near/2 improv*) or (health near/2 educat*) or (health near/2 practic*)

# 19 (#14 or #15 or #16 or #17 or #18)

#20 MeSH descriptor: [Community Health Nursing] explode all trees

#21 MeSH descriptor: [Education, Nursing] explode all trees

#22 MeSH descriptor: [Nurses] explode all trees

#23 MeSH descriptor: [Midwifery] explode all trees

#24 MeSH descriptor: [Allied Health Personnel] explode all trees

#25 MeSH descriptor: [Primary Health Care] explode all trees

#26 MeSH descriptor: [Community Health Workers] explode all trees

#27 nurs* near/2 educat*) or (nurs* near/2 train*) or (nurs* near/2 practic*) or (midwi* near/2 educat*) or (midwi* near/2 practic*) or (midwi* near/2 train*)

#28 (health visit*) or (home visit*) or (healthcare work*)

# 29 ( #20 or #21 or #22 or #23 or #24 or #25 or #26 or #27 or #28)

# 30 (#13 and #19 and #29)

**Database: CINHAL via EBSCO**

**Search strategy:**

S1 MH "oral health"

S2 MH "oral hygiene"

S3 MH "dental care"

S4 MH "mouth care"

S5 MH "dental caries"

S6 oral N2 (health or care or hygiene)

S7 dental N2 (care or health or hygiene)

S8 mouth N2 (care or health or hygiene)

S9 teeth N2 (care or hygiene or health)

S10 S1 OR S2 OR S3 OR S4 OR S5 OR S6 OR S7 OR S8 OR S9

S11 MH "health promotion"

S12 MH "oral health education"

S13 MH "preventive dentistry"

S14 ((oral health N2 (educat* or advice or promot* or improv*)

S15 ((dental health N2 (educat* or promot* or improv* or advice)

S16 ((prevent* N2 (dent* or oral)

S17 S11 OR S12 OR S13 OR S14 OR S15 OR S16

S18 MH "nurses"

S19 MH "midwifery"

S20 ((nurs* N2 (educat* or train* or practic*)

S 21 ((midwi* N2 (educat* or train* or practic*)

S22 health visit*

S23 home visit*

S24 healthcare work*

S25 S18 OR S19 OR S20 OR S21 OR S22 OR S23 OR S24

S26 S10 AND S17 AND S25

**Database: COS Conference Paper Index via PROQUEST**

**Search Strategy:**

(oral health AND nursing) OR (oral health AND midwifery) OR (dental care AND nurses) OR (dental care AND health visitors) OR (oral health pregnancy AND nurses) OR (school nurses AND moral health) OR (dental caries AND nurses) OR (periodontal diseases AND nurses) OR (oral health education AND nurses) OR (dental health education AND community health workers) OR (oral health AND health visitors)

**Database: ProQuest Dissertation and Theses A&I**

**Search Strategy:**

("oral hygiene child*" OR "dental care" OR "oral hygiene" OR "oral health child*" OR "dental caries child*" OR "dental care child*" OR "dental health child*") AND ("dental health promot*" OR "dental health educat*" OR "oral health educat*") AND ("nursing" OR "nurs*" OR midwives OR "health visitors" OR "home visitors")
